# Being targeted as a “severely overweight pregnant woman” —A qualitative interview study

**DOI:** 10.1111/hex.12681

**Published:** 2018-04-06

**Authors:** Drude S. Lauridsen, Peter Sandøe, Lotte Holm

**Affiliations:** ^1^ Department of Food and Resource Economics University of Copenhagen Frederiksberg Denmark; ^2^ Department of Food and Resource Economics Department of Veterinary and Animal Sciences University of Copenhagen Frederiksberg Denmark

**Keywords:** obesity, pregnancy, prenatal care, qualitative, target group, weight stigma

## Abstract

**Background:**

Pregnant women with a body mass index (BMI) ≥ 30 kg/m^2^ have been targeted in health‐care systems in many western countries as a high‐risk group. However, we have limited knowledge of the long‐term significance of this prenatal care policy.

**Objective:**

To investigate accounts women give of their experiences of being targeted as severely overweight during pregnancy when they look back at the intervention 4‐5 years later.

**Design:**

Interpretive analysis based on 21 semi‐structured interviews conducted 4‐5 years after the pregnancy with Danish mothers categorized as having a pre‐pregnancy BMI ≥ 30.

**Findings:**

In the women's retrospective accounts three phases were identified and separated: (i) Being identified as a “severely overweight pregnant woman.” The women differed over whether they accepted this categorization, but all believed that an approach based on weight was acceptable. (ii) Encounters with health‐care professionals. The women differed here: some reported no negative experiences; others reported experiences of prejudice and silence. (iii) Reflections on long‐term outcomes. Most women reported that the interventions during their pregnancies did not lead to any lasting lifestyle change. The women disagreed over whether, in principle, pregnancy was a suitable time to be targeted.

**Discussion and conclusion:**

Our study illustrates the importance of critically considering whether pregnancy is a suitable window of opportunity for obesity prevention, and shows that women's experiences should be examined in relation to each phase of intervention. More interdisciplinary studies are needed to map potential benefits and other consequences over the short‐ and long‐term.

## 1 INTRODUCTION

Over the last two decades, obesity in pregnancy has come to be framed as a major health problem. Studies show that during pregnancy women with obesity have a higher risk of health problems such as gestational diabetes and pre‐eclampsia,^1^ and are more likely to present with birth complications^2^ and foetal and neonatal risks.^3^, ^4^ Maternal obesity is also associated with increased birth weight,^1^ childhood obesity and later adult obesity.^5^, ^6^ Although evidence on the most effective way to intervene to reduce these risks is currently inconclusive, prenatal care policies in most western countries target pregnant women with obesity as a high‐risk group in selective health interventions.^7^ In Denmark, prenatal care guidelines in 2009 introduced a number of new initiatives aimed at pregnant women with a body mass index (BMI) ≥ 30 kg/m^2^: Expectant mothers were advised to restrict their weight gain to 6‐9 kg, maintain a healthy diet, and exercise more than 30 minutes a day.^8^

The category “severely overweight pregnant woman” denotes a relatively new target group in prenatal care. Many assumptions continue to be made about the potential benefits of intervening in pregnancy. In a public health perspective, pregnancy is considered an optimal time to intervene, as it is theorized as a “teachable moment” in which women are aware they are at risk, are motivated to change their lifestyle to protect their foetuses, and are changing their social identity, becoming a mother and a role model to their child.^9^ It seems likely, however, that interventions in a life event as significant and complex as pregnancy could result in unintended consequences.

Targeting women on the basis of their excess weight is sensitive given the long history of obesity stigma in western societies.^10^, ^11^ Stigma is here understood as a visible bodily mark that discredits the individual as different and discounted,^12^ because it is associated with negative stereotyping—in this case of overweight people as lazy, less competent and lacking in self‐discipline.^11^, ^13^ Being identified as “at risk” and part of a target group may lead people to self‐identify with the category, but external identification does not necessarily correspond with self‐identification or influence the behaviour of the people targeted.^14^, ^15^ While some women, when they are categorized as such, may not object to being seen as a “severely overweight pregnant woman,” others will not self‐identify as severely overweight or as “at risk,” and the targeting may then cause negative experiences.

Recent studies have highlighted the need to take women's experiences with obesity in pregnancy into account in order to improve prenatal care.^7^ The studies present contradictory results, however. Some indicate that women do not mind being approached on the basis of their weight and agree to participate in interventions to improve their own and their child's health.^16^ Others suggest that women find it embarrassing and humiliating to be singled out because of their weight,^17^ and that this can lead to a refusal to participate.^18^, ^19^ Studies have also shown that women living with obesity experience stigmatization from health‐care professionals during pregnancy,^17^, ^20^, ^21^, ^22^, ^23^ leading to feelings of frustration, shame and guilt.^21^ While these studies have provided a much‐needed perspective which may help to improve prenatal care, they are based on data gathered during pregnancy or shortly afterwards. However, as interventions during pregnancy are also assumed to have long‐term potential, studies of long‐term significance are needed.

In this paper, we therefore investigate women's retrospective accounts of their experience of being targeted in health interventions applying a pre‐pregnancy threshold BMI ≥ 30. Our research question was: *When looking back, what did the women experience when they were invited to take part in an intervention project focusing on severely overweight pregnant women?*

The women interviewed were 4‐5 years post‐pregnancy. The phrase “severely overweight pregnant woman” is an English translation of the term used within Danish health services to describe women in prenatal care with a BMI ≥ 30.^8^ We realize language here is a sensitive issue, and that the appropriate terms to describe people living with obesity are a matter of some debate.^24^ In using the phrase “severely overweight pregnant woman,” we have retained the quotation marks throughout as a reminder that the category is one externally constructed at a policy and science level and not one originating from a lay perspective.

In the paper, selective health promotions are analysed as processes involving a phase of identification, a phase of interaction in which health‐promotion efforts are made, and a phase of desired or unintended outcomes. The aim is to provide a better understanding of the long‐term significance of the identification, and the possible stigmatization women may have experienced during interventions, and to critically investigate the notion of pregnancy as a teachable moment.

## 2 METHOD

The study investigates mothers’ recalled experiences. Focusing on the mothers’ own perspectives, we conducted semi‐structured interviews,^25^ and used an interpretive reading approach^26^ to analyse their recollections. We did not assume that the recollections reflected events exactly as they occurred at the time. We recognized that the women's accounts were subjective recollections filtered and shaped to a greater or lesser extent by subsequent events in the women's lives. Permission for the study was granted by the Danish Data Protection Agency and the Research Ethics Committee for SCIENCE and SUND at the University of Copenhagen.

### 2.1 Recruitment

The women were recruited for the study from a convenience sample of mothers who, as well as being targeted for the standard prenatal care given to women with obesity, had participated in three scientific studies as a result of having pre‐pregnancy BMI ≥ 30. The first study was a three‐armed lifestyle intervention aiming to reduce weight‐gain during pregnancy.^27^ In this study, one group received dietary advice and a pedometer with advice to walk 11 000 steps a day. Another received the pedometer alone, and a control group received the standard prenatal care for women with BMI ≥ 30, which included one meeting with a dietician. The two subsequent studies did not focus on maternal diet or exercise. The second aimed to prolong the women's breast‐feeding^28^ and the third tracked the development of the children^29^ to the age of 3 years.

The women in this study were recruited by randomized quota sampling based on three educational levels. The group had already been selected in previous studies against criteria of fluency in Danish, a singleton pregnancy, and a normal scan at 11‐14 weeks. The women were first contacted via a letter informing them about the study and then later by telephone. In total 40 women were approached; 21 agreed to participate. The typical reason for declining to participate was that they had already invested considerable time in research projects. All women were guaranteed confidentiality and anonymity.

### 2.2 Data collection

The material reported here consists of 21 semi‐structured interviews of 56‐109 minutes conducted at the informant's home or the university (one interview was held by telephone) in the period December 2015‐April 2016. The interviews were in two parts. The first, involving the mother, focused on the pregnancy. In the second, both parents were interviewed about food, parenting, and child weight maintenance. In this article, we refer only to material from the first part. All interviews were conducted by the first author. After saturation was reached at the 18th interview, three additional interviews were conducted to ensure that no new topics were raised. The semi‐structured interview guide invited the women to talk openly about their experiences, and probed for more detail and reflection on the following themes: the experience of being pregnant, including pregnancy worries and complications, adherence to pregnancy advice, weight management during pregnancy, experience of prenatal care services and participation in a lifestyle intervention.

### 2.3 Data analysis

The interviews were transcribed verbatim, and after an initial read‐through, the material was thematically coded in NVivo 10 following guidelines proposed by Mason^26^. The themes were coded in an iterative process through which they were refined and subsequently arranged into phases. Throughout the analysis, we maintained an analytical openness to the possibility that a woman might have conflicting experiences and views on the same theme. In the subsequent interpretation of themes, quality was secured through researcher triangulation between the authors and by confronting the material with possible alternative interpretations. For the women, the scientific intervention and their prenatal care were intertwined parts of their pregnancy experience, and therefore both are analysed. In what follows the names are pseudonyms. Where parts of the interviewees’ remarks have been omitted for emphasis, this is clearly signalled with […].

## 3 FINDINGS

The interview group included first‐ and second‐time mothers with a range of educational affiliations and family arrangements (See Table 1). The findings are structured by the three phases being investigated, with each phase introducing connected themes: (i) How the women experienced targeting as “severely overweight pregnant women” and why they complied; (ii) experiences during pregnancy which, for some women, involved processing negative experiences and omission to mention weight and complications; (iii) the long‐term significance of being targeted as a person who is advised to change lifestyle. We found no clear relationships between informants’ experiences and their education, pregnancy number, or group type in the intervention, and therefore relationships of these kinds are not taken up in the analysis.

**Table 1:** Overview of informant characteristics

Name	Family relations	Pregnancy number	Intervention group in pregnancy	Education
Louise	Cohabitant	First time pregnant	Pedometer and dietarian	Long
Karen	Cohabitant	Third time pregnant	Pedometer	Long
Lone	Divorced	First time pregnant	Pedometer	Long
Sofie	Cohabitant	First time pregnant	Control	Long
Sandra	Cohabitant	Second time pregnant	Control	Long
Helle	Cohabitant	Second time pregnant	Pedometer and dietarian	Middle
Dorthe	Blended family	First time pregnant	Pedometer and dietarian	Short
Susanne	Cohabitant	First time pregnant	Pedometer and dietarian	Middle
Rita	Cohabitant	First time pregnant	Pedometer and dietarian	Short
Julie	Blended family	First time pregnant	Pedometer and dietarian	Middle
Michala	Cohabitant	Second time pregnant	Pedometer and dietarian	Middle
Mia	Cohabitant	First time pregnant	Pedometer and dietarian	Long
Solvej	Blended Family	First time pregnant	Pedometer	Short
Marianne	Cohabitant	First time pregnant	Pedometer	Short
Sigrid	Cohabitant	First time pregnant	Control	Long
Katrine	Cohabitant	First time pregnant	Control	Long
Stine	Divorced	Second time pregnant	Control	Long
Hanne	Cohabitant	Second time pregnant	Control	Short
Simone	Cohabitant	First time pregnant	Pedometer and dietarian	Long
Diana	Cohabitant	Second time pregnant	Pedometer and dietarian	Middle
Ida	Cohabitant	First time pregnant	Control	Middle

### 3.1 Phase 1: Being identified as a “severely overweight pregnant woman”

The first phase revolved around two subthemes: identification and willingness to participate. Being identified as having a high BMI in their pregnancy was something recalled by the women in differing ways, as the following shows. Most of the women were not surprised to be identified as having a high BMI, and comments such as “I knew that I weighed too much, so it wasn't like a big secret. So I honestly did not mind.” (Lone) were common. These women saw their status as individuals with high BMI as something that was already obvious. It was something they lived with every day, and they described themselves as having “a high BMI,” “weighing too much,” or “being big” (sometimes using the word “fat”). Another group of women—especially those with a BMI close to the lower threshold of 30 kg/m^2^—reported being a little surprised to be in the target group and referred to themselves as “a little overweight,” or “not over‐overweight”:

I was JUST on the brink of being able to join – at that time. But I remember and recollect that it was cool that something was done about this and investigated. (Simone)

Well, I remember I thought: wow, can it really be true that I'm that overweight? (Diana)

Looking back, the women did not feel that being targeted in pregnancy on the basis of weight had had any long‐term impact on the way they viewed their body weight. However, women in both groups sometimes referred the experience of identification in ambivalent terms, showing an awareness that overweight could be considered a negative stereotype. Still, these women acknowledged that their weight made them eligible for the intervention. The following remark illustrates this point:

[…] I would have liked to be a slim mother‐to‐be, walking around with a small football stomach. I would prefer that. So there was sort of a short moment where I felt sort of like: Oh, so I am a bit like trailer trash! No, well, sorry for the expression – it sounds arrogant – but sort of the kind of person who can't manage to eat properly, and exercise, and eats chips and béarnaise every day. […] Again it is natural that they ask me, and it is something that there is focus on, so I remember I thought: Well, but if it can help me. (Ida)

Despite the ambivalent accounts of targeting, all of the women thought it was acceptable to be approached, as they perceived the intervention as an offer of an extra service. Many women gave more than one reason for joining the intervention. The reasons were personal and altruistic, and sometimes both:

It is sort of the same reason as being a blood donor, or any kind of donor, right? If I want some myself I also have to give, right? (Sandra)

Some women reflected that being identified as being in the risk group was not something that had induced shame in them or made them feel like they had done wrong in some way, as they had not felt subjectively targeted:

I haven't felt targeted‐targeted […] You know there is a connection with gestational diabetes, and that has significance for both me and the child. I haven't felt sort of, how to put it, subjectively targeted, or outed. It has more been like, well, you can't run away from it. (Karen)

As this remark illustrates, for some women the targeting in itself was not problematic at the time, nor was it seen that way subsequently. This was because the women had internalized that excess weight in pregnancy could be a health risk. However, some women did remember feeling a little uncomfortable about being targeted or recalled being ambivalent at the time. During the interviews, these women reflected that while being targeted as individuals with a high BMI was somewhat intrusive, they nevertheless recognized that it was to the benefit of themselves and their children, and therefore acceptable.

### 3.2 Phase 2: Encounters with health‐care professionals

In the interviews, we asked the women to reflect on the focus on weight in their encounters with personnel in the research intervention and health‐care professionals in prenatal care. The women told various stories, some reporting no negative experiences, others describing experiences of direct weight stigmatization and some referring to episodes of awkward silence. The majority of women did not recall any negative experiences with health‐care professionals or scientific personnel.

Many of those negative stories you hear about how overweight people are provoked when they are in the health system—I have never experienced it. I've never had that feeling. I feel that I have always been treated well and had some sensible talks. (Helle)

The women who reported no negative experiences often explained that they had been treated like any other pregnant woman—although, of course, they had received extra services.

However, it also transpired in the interviews that some women had experienced what they recalled as incidents of prejudice and overtly negative comments. Interestingly, some of these women had explicitly also said that they had no negative experiences. Examples of prejudice towards the women included: being treated as less intelligent; staff assuming they did not know the general health advice; and their concerns not being taken seriously. Such events made the women feel they were seen as anonymous members of a category, not individuals with specific capabilities, challenges and needs. Examples of more overtly negative comments were as follows: being told that the single reason for feeling unwell during pregnancy was obesity, being scolded for gaining too much weight and being informed that their body weight made it difficult to examine them. Speaking about such experiences, the women often expressed anger, embarrassment and disbelief:

Well she said, sort of almost directly, that it was really impossible to scan me because I was so big […] I'm not the first they met out there who is overweight. And I'm certainly not the last. And I am not the largest they have seen either. So I just could not understand why she should scold me like that. But I think she had a bad day. We have written it off as that. (Marianne)

When reflecting on their negative experiences, the women employed different strategies. While they explicitly described their experience as a matter of being treated in ways that were wrong and unjustifiable, they had also, in retrospect, processed the events, either by offering excuses (eg the “bad day” mentioned above) or by portraying the event or members of staff as absurd:

[…] I actually changed midwife, as she asked me to only eat carrots for the rest of the pregnancy at week 12. Which I thought was an annoying response [laughs]. (Sigrid)

Some mothers felt that weight and weight‐gain restriction was referred to constantly. Others mentioned that doctors and midwives seemed afraid to broach these subjects. The women said that although it is never easy to have your “body flaws” pointed out, it could also become awkward if weight was not mentioned at all. While some women remembered doctors and midwives mentioning possible weight‐related complications in relation to birth and pregnancy, others had experienced silence on the matter, with such complications never being mentioned by their doctors and midwives. Instead, some women had found information for themselves, either online or by consulting books for expectant mothers. Others, quite consistently, could not recall being given, or seeing, information about weight‐related issues.

In their retrospective accounts, interviewees sometimes reflected on the most suitable time to inform women of health risks, and what information to provide. Some women thought the little information they were given was satisfactory. Others felt they should have been offered more. The women's comprehension of the dilemma of being exposed to too much or too little information is exemplified in the following remarks. In them, Ida reasons that although the mention of complications during pregnancy could cause unnecessary anxiety, it is necessary to address the issues to ensure women are not unaware of the risks in pregnancy:

When you are pregnant and overweight […], well, all the information about what can go wrong when you are overweight, how positively does that contribute to the process? […] On the other hand, you have to be informed about the risks: ‘Look, the fact is you're weighing 20 kilos too much, and, well, it is a fact that with overweight there is a greater risk for this and this and this, and you have to know that.’ (Ida)

Some women also touched on the awkwardness and frustration of being given information at a time when they were not able to use it due to circumstances they could not change.

### 3.3 Phase 3: Reflections on the long‐term outcome

In the women's retrospective accounts of being targeted as individuals who needed to change their lifestyle, the two important themes were barriers to the long‐term significance of the intervention, and the notion of pregnancy as a window of opportunity for lifestyle change. Reflecting on the targeting, only a few of the women remembered the intervention as something that had initiated a long‐term lifestyle change. For some, the intervention led to some changes during their pregnancy which they hoped had improved their child's health. However, these changes had not been maintained after childbirth. Two of the women, while they had not experienced any substantial life change, still counted their steps each day and thought about the dietary advice they had been given. The reasons given to explain the difficulty of making lifestyle changes were that the intervention was too short to alter deep‐rooted family habits; that changes were difficult to maintain when the child was born; and that families had to reconfigure everyday life to match the needs of a baby and working life:

I thought that it went really well during pregnancy, and I also thought it went well until I started at my job. It is very hard to use so much time to focus on your body and on food when you have a full‐time job, your partner too, and there is a child. So, it would have been better if it had started ten years earlier. There's no doubt about that. (Susanne)

Several women described how they felt it was difficult to prioritize their own well‐being and health post‐natally. In pregnancy, the health of the unborn child had helped them to stay motivated, but now they could care for their child's health without caring for themselves.

The women expressed differing opinions about the idea that pregnancy was an opportune time to initiate a lifestyle change. Some said that pregnancy was a good time, as women would want to protect and provide a healthy future for their child, and a time when other habits were revised in the light of prenatal advice: “It is definitely the most optimal time to target a mother” (Mia). Others said that pregnancy was a difficult time for the mother to make changes to her lifestyle, referring to the bodily, psychological and relational barriers that made such change difficult. These barriers included nausea and appetite issues which made changing diet feel impossible, physical complications which sometimes made exercise painful and difficult, and lack of time and spousal support. It was also mentioned that pregnancy “is a vulnerable time […] filled with hormones and worries” (Marianne) during which women can be made to feel guilty and lifestyle change is an overwhelming task. These reflections led several women to say that interventions designed to change their lifestyle would be more effective before, or after, pregnancy. As Sigrid explained, while pregnancy seems like a logical time, it is also a time when many other things are happening to a woman's mind and body:

If you just think about it logically, that you are carrying a baby and that is of course the time to be at your very healthiest. Then I just think that too much is going on, so that it is sometimes hard to, well, start it up then. (Sigrid)

A third response was that pregnancy is in general no better, or worse, than other points in life to initiate long‐term lifestyle change, as it is a very individual question of timing and motivation. Although most of the women did not consider that the health intervention had changed anything for them, almost all agreed that support for changes in diet and exercise during pregnancy should be provided for those who wanted it.

## 4 DISCUSSION

In this study, we investigated women's retrospective accounts, given 4‐5 years after the intervention, of their experience of being singled out in health interventions targeting those with a pre‐pregnancy BMI ≥ 30. The study contributes to our understanding of the long‐term significance, from the mother's perspective, of three phases of targeting: the initial targeting, encounters with health professionals, and the long‐term outcome. In retrospect, the women did not for the most part confirm that being approached on the basis of their weight had changed their self‐identification, but they differed in the way they looked back on their management of the experience of being treated in a stigmatizing way. The study also showed that there was little long‐term significance for the mothers when it came to lifestyle change. The women expressed various views about the suggestion that pregnancy was a suitable time to effect change.

Recollections of the first phase of targeting revolved around the themes of being identified as a target group based on weight, and agreeing to join interventions. In line with previous studies of women partaking in interventions and health‐care initiatives,^16^, ^30^ we found that being approached on the basis of high BMI was remembered, not as something negative, but rather as an offer of help, or as something undertaken through genuine concern about the women and their children. However, in our study, some women also recalled that, in being identified, they felt as though they were negatively stereotyped. This corroborates studies showing that pregnant women can feel a range of negative emotions when there is a focus on body size.^17^, ^21^ This discrepancy in our study suggests the category “severely overweight pregnant woman” is stigmatic—as it had negative connotations even for women who agreed to participate. However, although the categorization was unpleasant for some women at the time, it was not one to which the women had given much thought subsequently. While our study can be taken to accord with newer, explorative studies suggesting that midwives should not be afraid to approach women, and refer them for selective intervention, based on weight,^31^ it is still important to consider how this is done, and to anticipate that women are likely to react in different ways to the approach, given their differences in self‐identification, and as a result of the societal stigma associated with obesity.

In the second phase, where women were interacting with health‐care professionals, the management of negative comments and silences became important. Corroborating previous studies of pregnant women's experiences of weight stigmatisation,^17^, ^20^, ^21^, ^22^ some women in our study, even 4‐5 years later, remembered negative experiences that had made them feel bad. Further, these women were able to recount their experiences as encounters with prejudice and negative comment—that is as unjust treatment. They did not describe themselves as discredited individuals, and in this way, they protected themselves from a loss of self‐esteem.^13^ Interestingly, what some perceived as stigmatization was not experienced as such by other women. As a number of scholars have argued, it is important to educate health‐care professionals about women's perspectives and their own weight bias,^7^, ^20^, ^21^ but in addition, our study highlights that women can perceive and manage stigmatization in different ways depending on, for example, the situational interaction and their self‐identification.^12^, ^13^ Therefore, the education of health‐care professionals should equip them with an understanding of stigma as something that is negotiated in concrete interactions between the professional and the pregnant woman.

The reports in our study of health‐care professionals remaining silent on weight and its potential complications add to similar findings from previous studies,^20^, ^32^ as the failure to give information about risks and complications led some of the women to worry in the years after their pregnancy. Studies suggest that health‐care professionals who omit to mention weight‐related complications, or fail to give advice on how to handle these, may be afraid of offending women or simply embarrassed.^33^, ^34^, ^35^ However, these attitudes do not remove the stigma. The reticence can be seen as an expression of stigma—what Erving Goffman terms careful disattention.^12^ As the women are assigned a category during pregnancy which carries a stigma both in society and healthcare,^10^, ^11^, ^36^ silence leaves the task of managing obesity stigma, and learning about its potential health risks, on the women's shoulders. As several researchers have pointed out, a knowledge gap emerges if women with obesity are identified as a risk to themselves and their children in science, policy documents, and the media,^37^ and if some women are just not given information about the risks and complications, but are advised to restrict their weight gain. It has been pointed out that information should be given in a respectful^20^ and nuanced manner,^16^ especially as women are being informed about risks at a time when they find it hard to change their BMI.

Turning to the third phase, and long‐term outcomes, results from a recent study^16^ found pregnancy to be a teachable moment whose impact stretches beyond pregnancy and alters the lifestyle of the mother and subsequently her family. Our findings suggest this is perhaps too optimistic. The mothers in our study found that their participation in the intervention during pregnancy had very little long‐term significance: they had not achieved a lasting lifestyle change for themselves or their families. This finding is supported by a qualitative study from Sweden,^30^ which, to our knowledge, is the only other retrospective study on this topic. This, too, found that women had difficulty maintaining lifestyle change post‐natally as a consequence of work, stress, childcare and relationships with their partners.^30^ Like the researchers in the Swedish study, we acknowledge that interventions during pregnancy can lead to short‐term change, but we suggest that if the alleged window of opportunity is to be harnessed to full effect women will need continuing help post‐partum and to share responsibility with their partners and other family.^30^ However, it is important to stress that, at present, the notion of pregnancy as a window of opportunity rests on the assumption that mothers have sole responsibility for keeping children healthy^38^ and are able single‐handedly to change family routines. This assumption was not confirmed by the women's accounts in the present study. Our findings underline the importance of not only listening to the women's experiences to improve prenatal care, but letting women actively participate in the creation of interventions. Finally, intervention in pregnancy is not wholly positive. It can lead to maternal blame, and it can reproduce moral scrutiny of maternal behaviour and lead to further discrimination against women with obesity among the broader public.^39^

### 4.1 Limitations

Although the aim of qualitative studies like the one presented here is not generalization, it is important to address two limitations stemming from our recruitment of participants. First, the women who participated were a selected group that had already agreed to participate in several studies concerned with obesity. They could, therefore, be considered a group that were more able to accept being approached on the basis of their weight. Other women may feel it would be unacceptable to be approached on that basis, or they may be unable to process negative experiences in the way the women in this study did. A further limitation is that in five (ie approximately 25%) of the interviews the father was, at the mother's insistence, present for some of the interview on pregnancy experiences. This could have influenced the mother's stories, especially around issues of support from the partner with lifestyle change. However, some mothers mentioned lack of involvement despite paternal presence, and some fathers were supportive of the mothers’ stories.

## 5 CONCLUSION AND IMPLICATIONS

This retrospective investigation, conducted 4‐5 years after pregnancy, of women's natal and postnatal experience illustrates the importance of gaining knowledge of the long‐term significance of interventions aimed at pregnant women with obesity. The differences between the women's experiences, and the ambivalence felt by some women in this study about being targeted as “severely overweight pregnant women,” challenge scientists and policymakers when it comes to designing the best interventions. The challenge is to avoid assuming that women share characteristics and traits as a category, and to see each woman as an individual with specific resources, obstacles and needs. Those implementing health‐promotion strategies targeting pregnant women with obesity need to be sensitive to issues of self‐identification, and need also to think carefully about how to manage potentially stigmatizing encounters, and how to time lifestyle change in accordance with family circumstances. New types of intervention based on interdisciplinary research and co‐created lifestyle interventions are needed.

## CONFLICT OF INTEREST

Nothing to declare.
